# Second-language phoneme learning positively relates to voice recognition abilities in the native language: Evidence from behavior and brain potentials

**DOI:** 10.3389/fpsyg.2022.1008963

**Published:** 2022-10-12

**Authors:** Begoña Díaz, Gaël Cordero, Joyce Hoogendoorn, Nuria Sebastian-Galles

**Affiliations:** ^1^Department of Basic Sciences, Faculty of Medicine and Health Sciences, Universitat Internacional de Catalunya, Barcelona, Spain; ^2^Center for Brain and Cognition, University Pompeu Fabra, Barcelona, Spain

**Keywords:** phoneme learning, voice recognition, second-language, event-relate potentials, individual variability

## Abstract

Previous studies suggest a relationship between second-language learning and voice recognition processes, but the nature of such relation remains poorly understood. The present study investigates whether phoneme learning relates to voice recognition. A group of bilinguals that varied in their discrimination of a second-language phoneme contrast participated in this study. We assessed participants’ voice recognition skills in their native language at the behavioral and brain electrophysiological levels during a voice-avatar learning paradigm. Second-language phoneme discrimination positively correlated with behavioral and brain measures of voice recognition. At the electrophysiological level, correlations were present at two time windows and are interpreted within the dual-process model of recognition memory. The results are relevant to understanding the processes involved in language learning as they show a common variability for second-language phoneme and voice recognition processes.

## Introduction

The intercom rings. You walk over to the door and ask who it is. “Hey, it’s me.” Recognizing your friend, you buzz him in. Despite the apparent ambiguity of the answer, the message’s purpose – to identify himself as your friend – has been accomplished. This is because speech simultaneously conveys linguistic and paralinguistic information. Linguistic information refers to the phonemes that form a message whereas paralinguistic cues provide information about the speaker’s identity, emotional state, and social characteristics. Given that no two voices are identical; they can serve as an acoustic fingerprint that is highly valued in social interactions. Yet the uniqueness of voices poses challenges for speech perception: there is no one-to-one mapping between the perception of discrete phoneme categories and the acoustic properties of speech sounds across speakers (Peterson and Barney, 1952). Whereas native listeners of a language can easily deal with the acoustic variability of the speech signal, non-native listeners face the greater challenge of having to deal with this complexity without disposing of accurate phoneme representations. Recent findings of a bilingual advantage for voice recognition suggest that language learning and voice recognition are related abilities (Levi, 2018; Fecher and Johnson, 2019, 2022). However, the nature of such relationship is yet poorly understood. The present study investigates whether individual variability in discriminating second language (L2) phonemes relates to voice recognition abilities in a group of adult bilinguals.

A relation between phoneme and voice recognition is proposed by theories of speaker normalization which claim that voice-specific acoustic properties are mapped onto abstract, mental phoneme representations to achieve successful phoneme identification (for a review, see Johnson and Sjerps, 2021). Empirical findings support a relation between speech and voice recognition processes. Listeners perceive the same ambiguous stimuli as different vowels depending on the acoustic properties of the voices preceding sentences (Ladefoged and Broadbent, 1957; Sjerps and Smiljanić, 2013; Sjerps et al., 2019). In addition, speech comprehension improves when listening to familiar voices, as compared to unfamiliar voices, and when listening to a single voice, as compare to several alternating voices (Mullennix et al., 1989; Nygaard et al., 1994; Nygaard and Pisoni, 1998; Bradlow and Pisoni, 1999; Yonan and Sommers, 2000).

In the field of L2 learning, the association between phoneme and voice processes has been scarcely investigated. A relation of these two processes is suggested by findings of enhanced voice recognition abilities in bilinguals (Levi, 2018; Fecher and Johnson, 2019, 2022). Fecher and Johnson (2022) proposed that the origin of the so-called bilingual advantage for voice recognition is rooted on higher sensitivity to phonetic cues. They found that 9-month-old bilinguals were more accurate than their monolingual peers in discriminating voices speaking a language unfamiliar to the infants, whereas similar accuracy was found when the voices spoke the infants’ native language. The authors concluded that the bilingual advantage in voice recognition related to phoneme processes, as it was only apparent in the context of unfamiliar phonemes.

The present study investigates, for the first time, the hypothesis that L2 phoneme learning and voice recognition are related abilities. The participants were a group of 14 adult early-bilinguals similar in factors relevant for L2 learning such as L2 age of acquisition and L2 exposure, but yet showed individual variability in their overt discrimination of a L2 contrast consistently across phonological processes (sub-lexical and lexical). Participants were Spanish-Catalan bilinguals who were born and grew up in Catalonia (Spain), a bilingual region were both languages are co-official and coexist in most social environments. All participants were raised in monolingual Spanish families and were systematically exposed to the L2, Catalan, at the age of four at the latest, when they started mandatory bilingual schooling. This type of bilingual population has shown large individual variability to discriminate a L2 contrast, the Catalan-specific vowel contrast /e/−/ε/, which is considerably difficult for native Spanish speakers to discriminate as both members of the contrast are perceived as the only mid-front Spanish vowel /e/ (Pallier et al., 1997, 2001; Sebastian-Galles and Soto-Faraco, 1999; Bosch et al., 2000; Sebastian-Galles et al., 2005, 2006). For the present study, we selected participants from a larger sample (Schmitz et al., 2018) based on whether they exhibited consistent performance of L2 phoneme discrimination, nativelike or below native levels, across sub-lexical and lexical phonological processes in three behavioral tasks that evaluated the discrimination of the L2 contrast /e/−/ε/. The three behavioral tasks were an identification task, a gating task, and an auditory lexical decision task. These tasks have been previously employed to assess individual variability in L2 phoneme learning in bilingual populations (Sebastian-Galles and Baus, 2005; Díaz et al., 2008, 2012, 2016b; Schmitz et al., 2018). The identification task evaluated sub-lexical phonological processes and required participants to identify synthetic vowels from a continuum between /e/ and /ε/. The gating task tapped onto sub-lexical processing by evaluating the identification of naturally produced vowels on successive gates of minimal word pairs that differed in the L2 contrast /e/−/ε/. The auditory lexical decision task evaluated lexical processes as required participants to evaluate whether auditory stimuli were real L2 words. The experimental stimuli were words that contained the L2 vowel /e/ or /ε/ and non-words created by substituting in the words the critical vowel with the other vowel of the L2 phoneme contrast. The participants under study systematically succeeded or struggled with the discrimination of the L2 contrast in the three L2 behavioral tasks.

Participants were administered a Voice Recognition Task (VRT, adapted from Perrachione et al., 2011; Perea et al., 2014) that required learning the association between voices that spoke participants’ first language (L1) and avatars. We employed participants’ L1 to obtain a measure of voice recognition skills independent of non-native speech perception abilities. We registered the participants’ overt responses and brain event related potentials (ERPs), a measure previously employed to investigate voice processes. Past studies showed that voice recognition triggers positive effects from 300 ms after voice onset with variable scalp distribution across studies (Schweinberger, 2001; Zäske et al., 2014, 2018; Humble et al., 2019). In a second task, the Non-Word Association Task (NWAT), participants were asked to learn auditory non-words, enunciated by a single female voice, and avatars associations. Accurate performance of this task required learning the association between speech stimuli and faces, similar than in the VRT, but did not engage voice recognition processes. Thus, the NWAT served to evaluate participants’ capacity to learn audiovisual associations, an ability necessary to perform the VRT task but, *a priori*, unrelated to L2 phoneme learning.

If L2 phoneme learning and voice recognition are two related abilities, a positive correlation should be present between participants’ L2 phoneme discrimination and voice recognition accuracy at the behavioral level and brain electrophysiological, with positive correlations appearing latter than 300 ms. Moreover, no correlation should be present between participants’ L2 phoneme discrimination and the learning of non-words and avatars associations.

## Materials and methods

### Participants

A group of 14 Spanish-Catalan bilinguals participated in the study. All participants had similar language learning histories but differed in their final command of an L2 contrast. Participants were selected from an initial sample of 112 bilinguals studied by Schmitz et al. (2018). All participants in this initial sample lived all their lives in the Barcelona metropolitan area of Catalonia, where Spanish and Catalan are co-official languages. Even though the participants grew up in a bilingual society, their exposure to both languages was not equal during the initial years of their lives. All participants were raised in monolingual Spanish families and were not systematically exposed to the L2, Catalan, until the age of four, when they started bilingual mandatory schooling. All were graduate or undergraduate students and right-handed, as assessed by The Edinburgh Handedness Inventory (Oldfield, 1971). None of the participants reported a neurological or auditory problem nor had been diagnosed with a language disorder or learning disability. All participants were evaluated in their discrimination of an L2 contrast, the Catalan-specific vowel contrast /ε/−/e/, in three behavioral tasks with auditory stimuli: an identification task, a gating task, and a lexical decision task (Sebastian-Galles and Baus, 2005; Schmitz et al., 2018). The Catalan-specific vowel contrast /ε/−/e/ is considerably difficult for native Spanish speakers to discriminate (Sebastian-Galles and Soto-Faraco, 1999; Pallier et al., 2001; Sebastian-Galles et al., 2006; Sebastian-Galles and Díaz, 2012). The identification task presented a continuum of seven synthesized stimuli ranging from /e/ to /ε/ and participants were asked to identify for each stimulus whether it was the Catalan vowel /e/ or /ε/. The gating tasks consisted on presenting successive gates of minimal pairs that just differed in the Catalan vowels /e/ or /ε/. Participants task was to identify the word presented for each gate. The auditory lexical decision task consisted on the presentation of words that contained the Catalan vowel /e/ or /ε/ and non-words created by substituting in the words the critical vowel with the other vowel of the L2 phoneme contrast. Participants task was to determine whether the presented stimuli were real Catalan words. The identification and gating tasks evaluated sub-lexical processes with synthesized, the identification task, and naturally produced stimuli, the gating task. The lexical decision task evaluated the accuracy of lexical phonological processing.

Here, we investigated participants showing a consistent accuracy pattern, nativelike or below native level, across the sub-lexical and lexical phonological levels, that is, across the three tasks. From the initial population of 112 bilinguals, 23% (*n* = 25) of the participants scored within the range of native Catalan speakers (i.e., within 2.5 SD from the natives’ mean) in the three L2 tasks and 10% (*n* = 11) scored poorly in the three tasks (i.e., 3.5 SD below the natives’ mean). Fourteen participants were willing to participate in the present study: six participants (four females, mean age = 25.3 ± 1.2) that performed within the native range in all tasks and 8 (four females, mean age = 24.7 ± 1.7) that scored consistently poor in all L2 tasks. Participants reported an unbalanced exposure to the L1 and L2 during childhood (amount of exposure to the L1: 78.9%, amount of exposure to the L2: 19.7%, other languages: 1.4%), but a more balanced current use of each language (total use of L1: 54.3%, total use of L2: 39.1%, other languages: 6.6%). There were no differences in the exposure and use of the language between nativelike and below native performers (Table 1).

**Table 1:** Language variables and results of the L2 behavioral tasks as a function of the participant selection criterion: nativelike or below native performers.

	Nativelike performers: mean ± SD (range)	Below native performers: mean ± SD (range)	*t*-test, df = 12	Value of *p*
L1 exposure in childhood	81% ± 18 (60–100)	77% ± 18 (50–100)	<1	>0.05
L2 exposure in childhood	17% ± 18 (0–40)	21% ± 17 (0–50)	<1	>0.05
L1 current use	52% ± 16 (40–80)	56% ± 16 (20–70)	<1	>0.05
L2 current use	40% ± 14 (20–55)	38% ± 15 (25–70)	<1	>0.05
Identification task	0.94 ± 0.07 (0.83–1)	0.40 ± 0.23 (0.06–0.67)	5.37	<0.001
Gating task	0.99 ± 0.02 (0.94–1)	0.64 ± 0.16 (0.31–0.81)	7.22	<0.001
Lexical decision task: /e/ items	0.97 ± 0.01 (0.97–0.99)	0.71 ± 0.11 (0.50–0.83)	5.48	<0.001
Lexical decision task: /ε/ items	0.91 ± 0.04 (0.85–0.97)	0.78 ± 0.02 (0.76–0.81)	5.14	<0.001
L2 global score	0.95 ± 0.02 (0.93–0.98)	0.63 ± 0.06 (0.52–0.72)	11.65	<0.001

For each variable, independent sample *t*-tests were performed to compare the two groups. Note that the percentages for the language exposures and use for the childhood and current periods do not sum up 100% because of the presence of languages other than the L1, Spanish, and the L2, Catalan.

Participants scores in the L2 behavioral tasks ranged between 1 and 0.06 (mean = 0.63 ± 0.33) for the identification task, between 1 and 0.33 (mean = 0.79 ± 0.21) for the gating task, between 0.99 and 0.5 (mean = 0.83 ± 0.15) for the lexical decision task with items containing the vowel /e/, and between 0.97 and 0.76 (mean = 0.84 ± 0.07) for the lexical decision task with items containing the vowel /ε/. Table 1 shows the performance for each performers group and group comparisons. A combined L2 global score was obtained by averaging participants scores in the three L2 tasks (as in Sebastian-Galles et al., 2012). The combined scored ranged from 0.52 to 0.98 (mean = 0.77 ± 0.17) and was used in further analysis. None of the participants were professional musicians. Three participants (2 good and 1 poor L2 performers) reported playing an instrument since childhood and could be considered amateur musicians (Chartrand and Belin, 2006; Shaw, 2018).

### Stimuli

Auditory and visual stimuli were employed in two tasks. All auditory stimuli were in the participants’ L1, Spanish. For the voice recognition task (VRT), five female avatars were created using a free-to-use website.^1^ With permission of the authors, the auditory stimuli employed in Perea et al. (2014) were used. These stimuli consisted of ten Spanish sentences read by five female native Spanish speakers. For the non-word association task (NWAT), six new avatars were generated. A Spanish speaker, different from the ones employed for the VRT task, was recorded pronouncing six non-words: “veral,” “ceya,” “zobo,” “sulva,” “cutil,” “sodia” (from Carreiras et al., 1997). The intensity of all auditory stimuli was normalized by means of the software Praat (Boersma and van Heuven, 2001).

### Procedure

Experiments took place in an electrically shielded and sound-attenuated booth at the Neuroscience laboratory of the Center for Brain and Cognition (University Pompeu Fabra, Spain). The two tasks were controlled with Psychtoolbox 3.0.12 functions (Brainard, 1997), running on MATLAB 2015 (The MathWorks, Inc., MA, United States). Participants were comfortably seated in front of a 20-inch Samsung SyncMaster monitor while the auditory stimuli were presented *via* stereo Creative Inspire T10 speakers which flanked the screen. The sole language employed during the experiment was the participants’ L1, Spanish. All participants performed first the VRT and, right after, the NWRT.

#### Voice recognition task

The VRT consisted of three phases; a training phase, a short test phase with feedback, and a test phase. The training and test phases closely resembled the design employed in previous studies (Perrachione et al., 2011; Perea et al., 2014) with the difference that only voices speaking in the participants’ L1, Spanish, were used. We assessed behavioral responses for all three phases and we recorded participants’ electroencephalogram (EEG) during the test phase.

In the training phase, participants were trained to associate 5 avatars with their corresponding voices. Each trial consisted on the consecutive presentation of two avatar-voice pairs followed by the presentation of one sentence enunciated by one of the two voices just presented and participants were requested to provide the corresponding avatar. A trial started with the presentation of a black fixation point for 1 s followed by the sequential presentation of two avatars with an interstimulus interval (ISI) of 1 s during which a black fixation appeared on the screen. Simultaneous to the display of each avatar, a sentence spoken by the voice associated with that particular avatar was presented. Throughout each trial the same sentence was spoken by two voices. Subsequently, all five avatars were presented while one of the two previous auditory stimuli was repeated. A number [1, 2, 3, 4, 5] was displayed below each avatar. Participants had to identify the avatar that was associated with the voice by pressing the avatars number in a numeric keypad with their right index finger without the pressure of a time limit. Feedback concerning the accuracy of the answer was provided together with the image of the correct avatar. Half of the correct responses corresponded to the first avatar, and the other half to the second avatar. The following trial started 2 s after the participant provided their response. This training phase was composed of a total of 25 trials (five sentences × five avatars).

A short test phase with feedback was added to the design employed in previous studies (Perrachione et al., 2011; Perea et al., 2014) in order to enhance learning. This was done given that behavioral performance usually drops in EEG studies on account of the discomfort associated with this technique. The short test phase employed the same stimuli utilized in the training phase. A trial consisted in the presentation of one sentence and participants were asked to report the corresponding avatar. A trial started with the presentation of an auditory sentence while a black fixation point was displayed on a white background. After, the five avatars were displayed with their associated numbers displayed below them. Participants were asked to indicate which avatar was associated to the voice by pressing the corresponding key in the numeric keypad with their right index finger. No time limit to respond was imposed. Feedback was provided concerning the accuracy of their responses and the correct answer was provided. The short test phase was comprised of 25 trials (five sentences × five avatars). Right after, test phase commenced with the same experimental design than the short test phase with the exception that five new sentences were used and no feedback was provided. The test phase was comprised of 50 trials (five sentences × five avatars × two repetitions).

Eight stimuli lists were created with the constraint that the same voice could be presented, at most, in three consecutive trials and the same sentence could be, at most, in two consecutive trials. Each list had different avatar-voice pairs. The total task lasted ~20 min.

#### Non-word association task

The NWAT included a training phase and a test phase which sought to train and test participants on audiovisual associations. Auditory stimuli consisted of six non-words recorded by a native Spanish female speaker. In the training phase participants had to learn the association between each of the six non-words and its corresponding avatars. The training had 12 trials in which a nonword and its corresponding avatar were simultaneously presented followed by the presentation of a black fixation point for 1 s. Each non-word and avatar association was presented twice. The test phase employed the same stimuli as the training phase and was composed of 48 trials (eight repetitions of each nonword-avatar association). A test trial consisted of the presentation of a non-word while the screen displayed a black fixation point. Subsequently, the six avatars were presented on the screen with a number displayed below them (from 1 to 6). Participants indicated which avatar was associated to the nonword by pressing the corresponding key in the numeric keypad with their right index finger. No time constraint was given to respond. Following the response, a black fixation point was displayed on a white background for 2 s before the next trial began. Eight stimuli lists were created with the constraint that the same non-word could be presented, at most, in two consecutive trials. Different non-word-avatar pairings were established in each list. The total duration of this task was 5 min.

### Electrophysiological recording

The EEG was recorded from 64 tin actiCAP electrodes (Brain Products, Gliching, Germany) at positions Fp1, Fp2, AF7, AF3, AF4, AF8, F7, F3, F1, Fz, F2, F4, F8, FT9, FT7, FC5, FC3, FC1, FC2, FC4, FC6, FT8, FT10, T7, C5, C3, C1, Cz, C2, C4, C6, T8, TP9, TP7, CP5, CP3, CP1, CPz, CP2, CP4, CP6, TP8, TP10, P7, P5, P3, P1, Pz, P2, P4, P6, P8, PO3, POz, PO4, PO9, O1, Oz, O2, PO10 (according to the actiCAP 64-standard-2 placement system). Impedances were kept below 25 kOhm. To monitor eye-movements, the horizontal electrooculogram (EOG) was recorded with an electrode attached to the outer canthi of the right eye while for vertical EOG, an electrode was placed below the right eye. EEG activity was registered with a sampling rate of 500 Hz and by employing the left mastoid as reference.

### ERP data analysis

The EEG data was pre-processed with Brain Vision2 (Brain Products, Gliching, Germany). An offline band-pass filter of 0.1–50 Hz and a 50 Hz notch filter (both with a slope of 12 dB/oct) were applied to the data. Flat or contaminated channels due to electrode failure were excluded and reconstructed by means of the topographic interpolation tool included in Brain Vision2. Eye-movement and blinking were corrected using the ocular independent component analyses (Ocular ICA) implemented in Brain Vision2. The signal was rereferenced to the right and left mastoids. We automatically rejected offline those EEG epochs in which any channel either exceeded ±100 μV, had an activity below 0.5 μV, or showed voltage step/sampling above 50 μV within intervals of 100 ms. The epochs were time-locked to the onset of the test sentences and were 1,600 ms long, including a pre-stimulus baseline of 100 ms. Epochs of all test trials, regardless of the correctness of the response, were averaged separately for each participant. This approach is consistent with ERP studies that aim to characterize the brain activity that leads to different degrees of proficiency in a task (Weber-Fox and Neville, 1996; Sebastian-Galles et al., 2006; Alemán Bañón et al., 2012; Díaz et al., 2016a; Zawiszewski and Laka, 2020; Gabriele et al., 2021).

The temporal windows of interest were determined by an electrode-level analysis. For each electrode, we ran right-tailed Pearson correlations in successive time windows of 20 ms between the L2 global score and the EEG amplitudes during the VRT using Matlab (R2021a, Statistical and Machine Learning Toolbox version 12.1, The MathWorks, Inc., MA, United States). Following previous studies, we controlled for false positives that can occur when a large number of statistical comparisons are performed by considering effects present for at least two consecutive intervals and at least for four electrodes (Gunter et al., 1997, 2000; Hahne and Friederici, 1999; Díaz et al., 2011, 2016a). The time windows revealed by the electrode-level analysis were further analyzed at the scalp-level for four region of interest (ROI): frontal left (F1, F3, F7, FC1, FC3, FC5, FT7), frontal right (F2, F4, F8, FC2, FC4, FC6, FT8), posterior left (CP1, CP3, CP5, P1, P3, P5, TP7), and posterior right (CP2, CP4, CP6, P2, P4, P6, TP8). We ran right-tailed Pearson correlations between the L2 global score and the mean EEG amplitudes of each ROI. We report the *p*-values of the correlations together with the correlation coefficients (*r*; absolute values of 0.1, 0.3, and 0.5 indicate small, medium, and large effect sizes, respectively). We assessed for laterality effects by comparing statistically the significant correlations revealed at each hemisphere and time window by means of Matlab (function corr_rtest, Matlab File Exchange). For the sake of completeness, the exact same analysis was performed between participants’ accuracy in the NWAT and the EEG amplitudes during the VRT.

## Results

### Behavioral data

**Figure 1:**
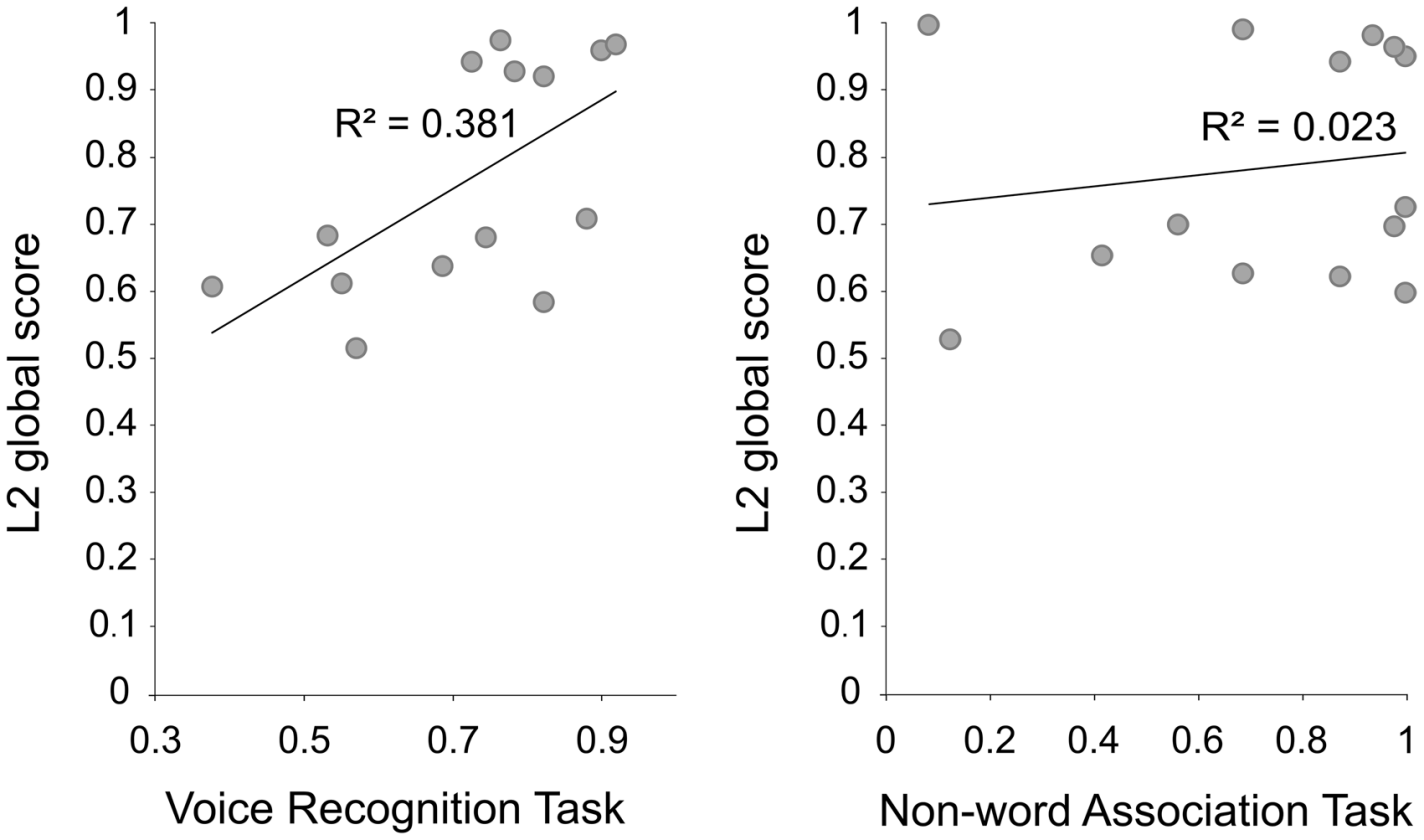
The scatter plots depict the relationship between the L2 global score and behavioral accuracy in the voice recognition task (*r* = 0.61, *p* = 0.009), and in the non-word association task (*r* = 0.15, *p* > 0.05).

Data was analyzed with Matlab (R2021a, Statistical and Machine Learning Toolbox version 12.1, The MathWorks, Inc., MA, United States). Participants performed similarly in the VRT (mean accuracy rate = 0.73 ± 0.16) and NWAT (mean accuracy rate = 0.72 ± 0.32; paired-samples *t*-test: *t* (13) < 1). As expected, the L2 global score positively correlated with performance of the VRT (*r* = 0.61, *p* = 0.009) but not with the performance of the NWAT (*r* = 0.15, *p* > 0.05; Figure 1).

### ERP data for the voice recognition task

**Figure 2:**
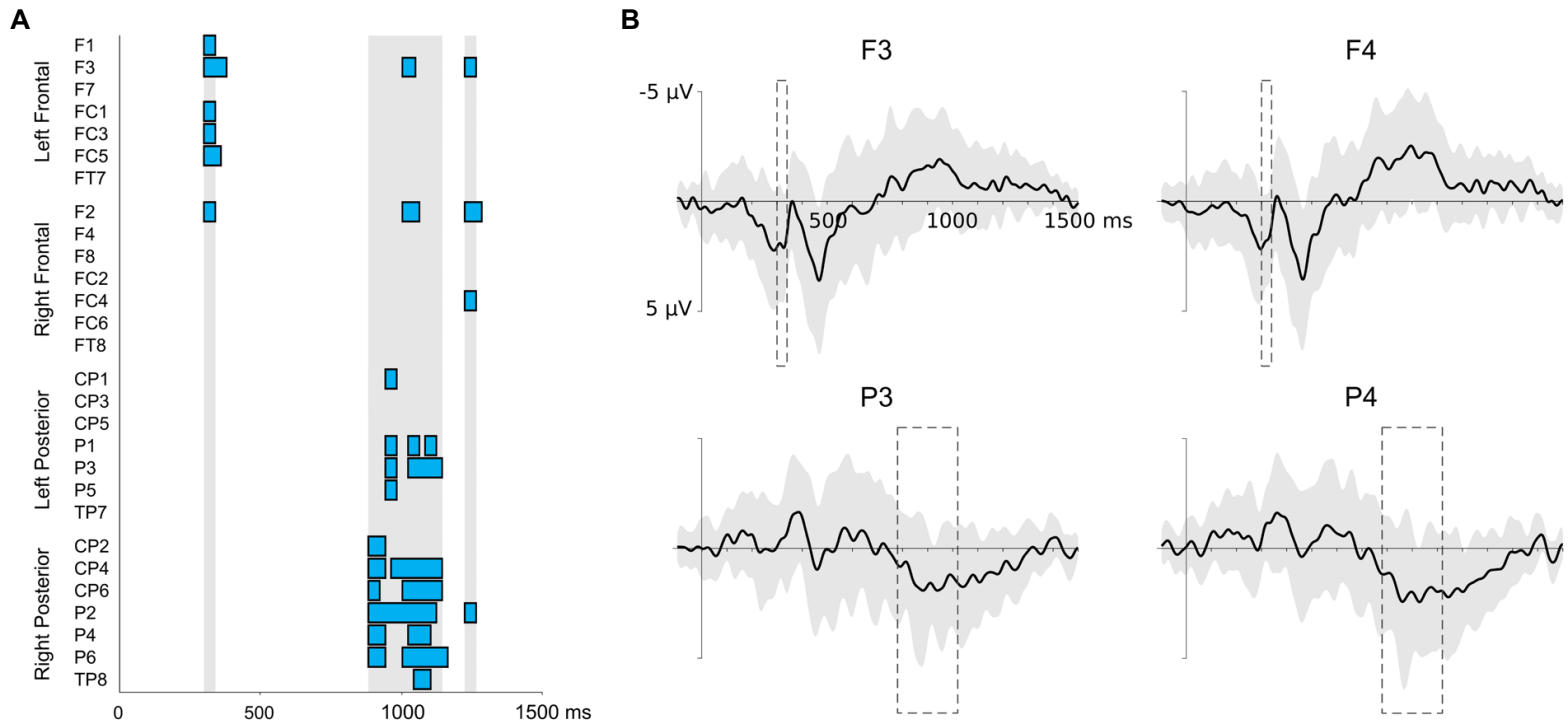
Results of the correlations run in successive time windows of 20 ms between the L2 global score and the EEG amplitudes during the VRT **(A)**. Electrodes are grouped into four ROIs (frontal left, frontal right, posterior left, and posterior right). The beginning of the epochs is time-locked to the onset of the sentences. For each electrode, significant correlations are indicated by blue boxes. The vertical light gray areas indicate the time windows included in scalp-level analysis. **(B)** Grand-average ERP (±1 standard deviation represented by the light gray area) of four representative electrodes during the VRT. Time windows where significant correlations between the L2 global score and the EEG amplitudes during the VRT were present are indicated by the dash line boxes.

Figure 2 displays the onsets and durations of the ERP effects at the electrode-level in the analysis of successive 20 ms time windows and the grand average EEG waveforms at four representative electrodes of each ROI. The analysis at the electrode-level showed significant correlations between the L2 global score and the EEG activity during the VRT for three time windows: between 300 ms and 340 ms, between 880 ms and 1,140 ms, and between 1,220 ms and 1,260 ms. There were no significant correlations at the electrode-level between participants’ accuracy in the NWAT and the EEG during the VRT.

**Figure 3:**
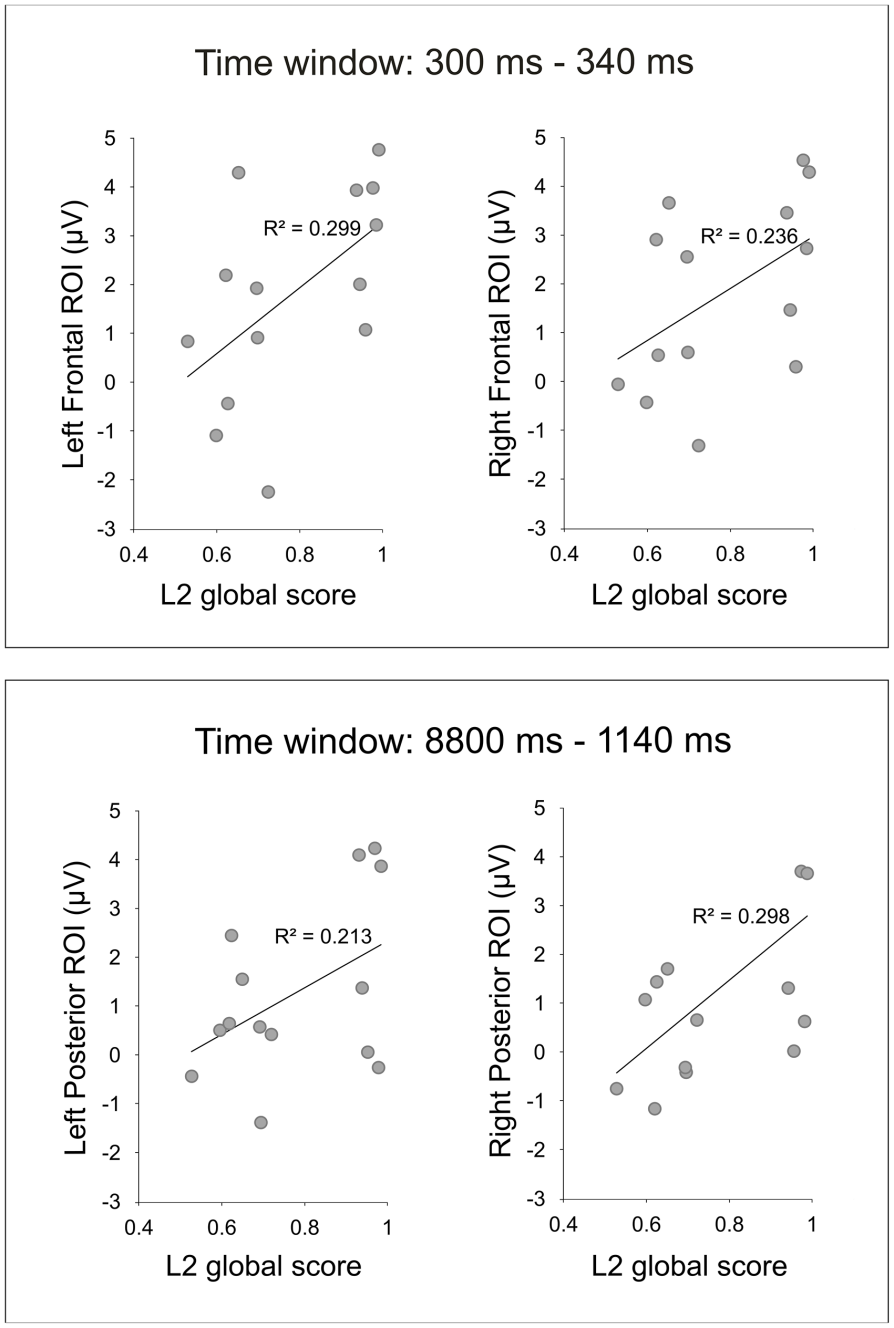
Scatter plots depict the significant positive correlations revealed by the scalp-level analysis between the mean EEG amplitudes during the VRT and the L2 global score.

The analysis at the scalp-level revealed reliable correlations between the L2 global score and the EEG during the VRT for two time windows, 300–340 ms and 880–1,140 ms (Table 2). For the first time window between 300 and 340 ms, the L2 global score and the EEG amplitudes correlated positively at right and left frontal regions (Figure 3). There were no laterality effects, the strength of the correlations was similar (*p* = 0.845). For the second time window between 880 and 1,140 ms, the two measures significantly correlated at right and left posterior regions (Figure 3). Again, there was no laterality effect, the two correlations did not differ significantly (*p* = 0.789). For the third time window between 1,220 and 1,260 ms, there was no significant correlation (the correlation only approached significance at the frontal right and posterior right regions).

**Table 2:** Results of the correlation analysis at the scalp-level between the L2 global score and the EEG for the VRT.

	Time windows					
	300–340 ms		880–1,140 ms		1,220–1,260 ms	
Regions of interest	*r*	*p*	*r*	*p*	*r*	*p*
Right frontal	0.486	0.038^*^	0.324	0.129	0.414	0.070
Left frontal	0.547	0.021^*^	0.197	0.249	0.265	0.179
Right posterior	0.233	0.211	0.546	0.021^*^	0.442	0.056
Left posterior	0.421	0.066	0.461	0.048^*^	0.295	0.152

The table shows the results of the Pearson correlations (*r* coefficients) and statistical significance (*p*-values) between the L2 global score and the EEG during the VRT as a function of the region of interest and the time window determined at the electrode-level. ^*^Significant correlations, *p* < 0.05.

Table 2 shows the results of the Pearson correlations (*r* coefficients) and statistical significance (*p*-values) between the L2 global score and the EEG amplitudes during the VRT as a function of the region of interest and the time window determined at the electrode-level.

## Discussion

The present study provides supporting evidence for the relation between phoneme and voice recognition abilities offered by Fecher and Johnson (2022) to explain the bilingual advantage in voice recognition. Here, we found that the ability of a group of adult bilinguals to overtly discriminate a L2 phoneme contrast positively correlated with their L1 voice recognition abilities at the behavioral and brain electrophysiological levels. Yet, the origin of the relation in the present study is of a distinct nature than the one in Fecher and Johnson (2022). Different than in Fecher and Johnson (2022), where bilinguals infants were compared to monolinguals, the participants in the present study were all adult bilinguals who had similar language learning histories. Thus, the present variability in voice recognition cannot be attributed to bilingualism.

The association between L2 speech learning and L1 voice recognition abilities, *a priori*, two unrelated processes, can be understood through the lenses of speaker normalization theories, which propose that speaker voice invariants need to be identified to enable the discovery of speech invariants (for a review, see Johnson and Sjerps, 2021). The present findings support speaker normalization theories and extend their proposal to L2 language learning. Following this theory’s assumptions, one possible explanation of the present findings is that high accuracy in voice recognition provide a competitive edge to learn new phonemes. People with good voice recognition abilities may have a better ability to identify the stable acoustic properties related to voices which would enable them a higher accuracy in finding the cues that identify phonemes. Alternatively, speaker normalization theories would also lead to the explanation that accurate phoneme processes may lead to enhanced voice recognition abilities. Previous studies with early and late bilinguals revealed the existence of a general ability for phoneme discrimination regardless of phoneme familiarity (Díaz et al., 2008, 2016b). Hence, variability in discriminating phonemic changes within the speech signal may be the basis of individual differences in identifying the stable traits that characterize voices. Selecting between these two alternatives requires of further research as the present correlation study does not allow to establish a causal relation between the two processes.

The present correlation between L2 phoneme and voice recognition abilities did not seem to be mediated by differences in general-domain abilities engaged by the voice recognition task. We assessed participants ability to learn audiovisual associations, an ability necessary to perform the voice-avatar learning paradigm. Participants’ accuracy in learning non-word and avatar pairs did not relate to their L2 phoneme discrimination abilities. In addition, the lack of significant correlations between L2 phoneme discrimination and the electrophysiological activity to voice recognition at early latencies (<300 ms) at which auditory evoked potentials emerge, such as the N1 and P2 (Picton et al., 1974), can be taken as an indication that general-domain auditory analysis skills did not play a role in the present association between L2 phoneme and voice recognition abilities. Note, however, that cortical ERPs may not necessary capture the participants’ ability to make use of the acoustic information. The lack of effects for the NWAT might suggest that auditory perceptual abilities did not mediate the correlations between L2 phoneme discrimination and voice recognition abilities. Yet, performance of the NWAT relied not only on auditory, but also visual abilities and may not serve as an accurate test for auditory perception. It is thus feasible that auditory perceptual abilities, apart from voice recognition, contributed to the present findings.

The brain electrophysiological results showed a positive relation between L2 phoneme discrimination and voice recognition abilities at two distinct time windows, between 300 and 340 ms and between 880 and 1,140 ms. At each time window, the effects had a distinct scalp distribution: frontal during the first time window and posterior during the second one. The findings are in line with previous studies that reported positivities triggered by voice recognition 300 ms after stimuli onset (Schweinberger, 2001; Zäske et al., 2014, 2018; Humble et al., 2019). Yet, the distinct latency and scalp distribution of the two ERP effects in the present study suggest that they index distinct processes of recognition. This interpretation is in line with the dual-process model of recognition memory, which conceptualizes recognition as the results of two sequential processes: an automatic familiarity judgment followed by the effortful recollection of the properties of the stimuli (Yonelinas, 1994; Wixted, 2007). In agreement with this model, recognition of familiar stimuli triggers two positive ERP components during old/new tasks: a frontal positivity between 300 and 500 ms claimed to mirror the initial familiarity judgment and a parietal positivity from about 500 ms attributed to recollection from memory of the properties of the stimuli (for a review, see Rugg and Curran, 2007). The present ERP effects agree in latency and scalp distribution with those triggered by old/new tasks. Given the similarities, we interpret the present findings as a suggestion that individual variability in L2 phoneme discrimination relates to the initial familiarity judgment of voices and the subsequent intentional recollection from memory of the specific properties of the voice that matches the sensory input.

The present study reveals, for the first time, an association between L2 phoneme learning skills and L1 voice recognition abilities, at the behavioral and brain electrophysiological levels. These findings are relevant to understand the processes involved in language learning and contribute to the understanding of speech perception. In addition, the association between the two abilities support the view that voice recognition may be a suitable tool to predict the outcome of L2 learning before learning itself starts. Further research is needed to establish the causal relation between L2 phoneme learning and voice recognition.

## Data availability statement

The raw data supporting the conclusions of this article will be made available by the authors, without undue reservation.

## Ethics statement

The studies involving human participants were reviewed and approved by Clinical research ethics committee at the Parc de la Salut Mar. The patients/participants provided their written informed consent to participate in this study.

## Author contributions

BD, GC, JH, and NS-G contributed to conception and design of the study and wrote the first draft of the manuscript. BD, GC, and JH performed research and analyzed the data. All authors contributed to the article and approved the submitted version.

## Funding

This research was supported by grants from the Spanish Government (PID2019-106924GA-I00 and PID2021-123416NB-I00 financed by MCIN/AEI/10.13039/501100011033/FEDER, UE), the Catalan Government (SGR 2017-268 and ICREA [Catalan Institution for Research and Advanced Studies] Academia 2019 award), and an Universitat Internacional de Catalunya PhD grant.

## Conflict of interest

The authors declare that the research was conducted in the absence of any commercial or financial relationships that could be construed as a potential conflict of interest.

## Publisher’s note

All claims expressed in this article are solely those of the authors and do not necessarily represent those of their affiliated organizations, or those of the publisher, the editors and the reviewers. Any product that may be evaluated in this article, or claim that may be made by its manufacturer, is not guaranteed or endorsed by the publisher.

## Footnotes

^1^ www.crearunavatar.com
